# Application of Stem Cells Shows Antiinflammatory Effect in an Irradiated Random Pattern Flap Model

**DOI:** 10.3390/jpm14060554

**Published:** 2024-05-23

**Authors:** Wibke Müller-Seubert, Lena Fuchs, Raymund E. Horch, Luitpold Distel, Benjamin Frey, Isabell Renno, Ramona Erber, Andreas Arkudas

**Affiliations:** 1 Laboratory for Tissue Engineering and Regenerative Medicine, Department of Plastic and Hand Surgery, University Hospital Erlangen, Friedrich Alexander University Erlangen-Nuernberg (FAU), Krankenhausstr. 12, 91054 Erlangen, Germany; 2 Department of Radiation Oncology, University Hospital Erlangen, Friedrich Alexander University Erlangen-Nuernberg (FAU), Universitätsstr. 27, 91054 Erlangen, Germany; 3 Translational Radiobiology, Department of Radiation Oncology, University Hospital Erlangen Universitätsklinikum Erlangen, Friedrich Alexander University Erlangen-Nuernberg (FAU), Universitätsstr. 27, 91054 Erlangen, Germany; 4 Institute of Pathology, University Hospital Erlangen, Comprehensive Cancer Center Erlangen-EMN, Friedrich Alexander University Erlangen-Nuernberg (FAU), Krankenhausstraße 8-10, 91054 Erlangen, Germany

**Keywords:** ASC, MSC, HUVEC, irradiation, random pattern flaps

## Abstract

Background: In reconstructive surgery, local flaps might develop tissue necrosis or partial flap loss especially after previous irradiation, which may be necessary in many tumor entities. The application of stem cells seems promising to improve flap perfusion and might be a possible solution to optimize flap survival. Methods: Twenty rats received harvesting of bilateral random pattern fasciocutaneous flaps. The right flaps received 20 Gy ionizing radiation 4 weeks prior to the surgery, while the left flaps served as the non-irradiated control. After flap harvest, four different stem cell mixtures (5 × 10^6^ ASC, ASC-HUVEC, MSC, MSC-HUVEC) were applied under both right and left flaps using 1 mL fibrin glue as the delivery vehicle. Flap size and its necrotic area were examined clinically. Two weeks after the surgery, HE staining and immunohistochemical staining for CD68 and ERG, as well as PCR analysis (Interleukin 6, HIF-1α and VEGF), were performed. Results: Application of ASCs, ASCs-HUVECs and MSCs resulted in a lower number of CD68-stained cells compared to the no cell group. The expression of Hif1α was higher in the ASC group compared to those in the MSC and previously treated no cell groups. Treatment with MSCs and MSCs-HUVECs prevented shrinking of the flaps in this series. Conclusion: Application of ASCs, MSCs and ASCs-HUVECs was shown to have an antiinflammatory effect. Treatment with MSCs and MSCs-HUVECs can prevent early shrinking of the flaps.

## 1. Introduction

Defect reconstruction remains one of the main topics in plastic-reconstructive surgery. Many tumor entities, for example, in the head, neck or breast, require adjuvant radiotherapy, with possible negative side effects such as tissue fibrosis or non-healing ulcers [1]. Irradiation therapy delivers lethal doses of radiation to areas of malignancy to destroy cancer cells. As a consequence, the surrounding healthy cells are also exposed to lethal doses of radiation [2,3]. A negative side effect is the development of radiodermatitis with erythema, ulceration and pain, followed by chronic soft tissue atrophy, alopecia and stiffness [2]. Radiation-induced damage and inflammation often lead to chronic radiation-induced fibrosis within 4 to 12 months following therapy and may continue for years [2,3].

As a possible consequence, defect reconstruction using local flaps from the adjacent tissue or even free flaps might have a higher risk for wound healing disorders. Local flaps from the surrounding previously irradiated tissue might develop tissue necrosis or partial flap loss. 

Therefore, application of stem cells might be a possible solution. Topical application of a mixture of different stem cells such umbilical cord mesenchymal stem cells and other regenerative medicine products in a patient with an irradiated perineal wound after anal squamous cell cancer and flap failure resulted in wound healing after 79 days [3]. Application of conditioned mesenchymal stroma cells in severe local radiation injuries accelerated wound healing [4,5]. 

In general, adipose mesenchymal stem cells (ASCs) stimulate neovascularization and angiogenesis [6]. These effects have been used to treat critical limb ischemia in 15 patients and improved the clinical symptoms and showed formation of numerous vascular collateral networks after multiple intramuscular injections of ASCs [7]. Bone marrow-derived stem cells (MSCs) have the capacity to promote angiogenesis and decrease fibrosis [8,9]. Furthermore, they have shown to improve hind limb ischemia in a mouse model after intramuscular injection [10]. Intravenous application of MSCs in a rat brain ischemia/reperfusion model 7 days after ischemia resulted in a significant higher perfusion of the brain after 21 days compared to the control group without application of MSCs [11]. MSCs secrete factors that promote tissue neovascularization such as VEGF, IL-6 and IL-8 [11,12,13]. IL-6 has been shown to be highly expressed in both MSCs and ASCs when analyzing the cytokine expression profile [14,15]. Human umbilical vascular endothelial cells (HUVECs) have the ability to create robust microvascular networks in vivo and in vitro [16,17,18,19]. 

In a previous study, the influence of different irradiation regimes on flap perfusion in a random pattern rat model have been studied. It has been shown that preoperative fractional irradiation with a lower individual dose but a higher total dose has a more negative impact on flap perfusion compared to higher single stage irradiation [20,21]. Based on these findings, this study investigates the influence of different cells on preoperatively irradiated random pattern flaps. Here, the size of the necrotic area of the flaps, histological changes and the expression of different genes are analyzed in particular.

## 2. Materials and Methods

Twenty male RNU rats (age 16.4 ± 5.6 weeks (range 11–30 weeks); with a weight of 337 ± 48.8 g (range 244–440 g)) were operated in four different treatment groups. All rats received single-stage irradiation of the prospective area of the right flap with 20 Gy ionizing radiation (IR) four weeks before the operation. This study was authorized by the ethics committee of the government of Middle Franconia (RUF-55.2.2-2532-2-1275-15). A previously operated group (Lewis rats) that received 20 Gy preoperative irradiation but no cell application served as the control group [21].

The surgery and the irradiation procedures have already been described [20,21]. 

In brief, an orthovoltage X-ray device was used to perform the irradiation with a current of 20 mA and a voltage of 150 kV. For intramuscular anesthesia, ketamine (100 mg per kg) and medetomidine (0.2 mg per kg) were used. Rats were placed on their stomach and transported to the irradiation unit in a closed isolation cage, so that the rats were protected against pathogens. An area of 7 × 2 cm^2^ including the area of the prospective right flap was irradiated. Lead shields were used to cover the rest of the body of the rat to protect them from irradiation. 

**Figure 1:**
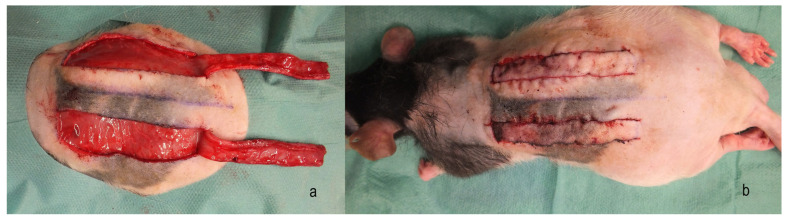
Harvesting of two modified McFarlane flaps (**a**) and after reinsertion of the flaps (**b**).

For surgery, isofluran was used for anesthesia and the animals received butorphanol (0.05–0.2 mg per kg) and meloxicam (1 mg per kg). Two modified McFarlane flaps that were based caudally were harvested from the rat’s back, measuring a length of 6 cm and a width of 1 cm. The flaps were placed parallel and 1 cm lateral to the spine, with their caudal base 1 cm cranial of the spina iliaca posterior superior (Figure 1). 

For harvesting, the incision was performed along their medial, lateral and cranial sides. After raising the flap, different cell mixtures were applied to the wound bed and the flap was relocated to its bed, and monofilament and polyfilament sutures were used for wound closing. Postoperative analgesia was performed using meloxicam. The rats received an antibiotic treatment with enrofloxacin (7.5 mg per kg) for 5 days. 

**Figure 2:**
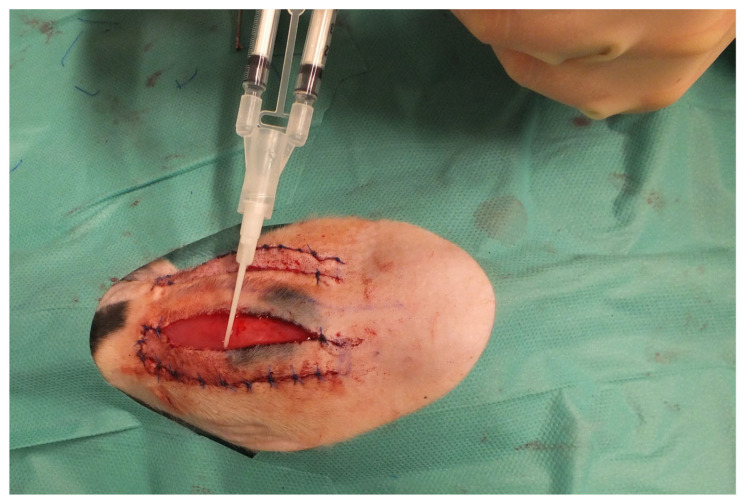
Application of the cells under the flaps using fibrin glue.

Four different cell mixtures were applied under both right and left flaps (Figure 2).

In total, 5 × 10^6^ cells were applied using 1 mL fibrin glue (Baxter, Deerfield, IL, USA) as the carrier substance under each flap. 

The left flaps served as the non-irradiated control (see all groups in Table 1).

In group one, ASCs (ASC/tert1, Evercyte GmbH, Vienna, Austria) were applied, in group two ASCs and HUVECs (HUVEC/tert2, Evercyte GmbH), in group three MSCs (BM-MSC/tert292, Evercyte GmbH) and in group four MSCs and HUVECs. ASCs were cultivated in EBM-2 medium (Lonza Group, Bensheim, Germany) and 2% fetal calf serum (FCS). HUVECs were cultivated in EBM-2 medium and 10% FCS. MSCs were cultivated in MesenCult-ACF Plus (Stemcell, Vancouver, BC, Canada) with 1% L-Glutamin. 

Standard clinical imaging to determine the visible necrotic area of the flaps and the flap size was performed directly after the operation and on the postoperative days 1, 3, 7, 10 and 14. Explantation of the flaps was performed 14 days postoperatively. Each flap was divided first longitudinally and divided into thirds. As previously described, the stainings were performed on the cranial lateral third (Table 2) and the PCR analyses on the cranial medial third [21]. 

For performing the staining, the flaps were embedded in such a manner that they could be evaluated in all layers from dorsal to basal [21]. Hematoxylin and eosin (H&E) staining and immunohistochemical stainings with CD68 (ED1 clone, Bio-Rad, Feldkrichen, Germany) as the pan-macrophage marker and ERG (clone EP111, Epitomics, Inc., Burlingame, CA, USA) for vessels were performed. PCR analyses were performed with previously designed primers for Interleukin 6, HIF-1α and VEGF [21]. GAPDH served as the housekeeping gene. All primers (Table 3) were purchased from the Sigma-Aldrich Corporation. PCR was performed in triplets of each specimen. 

The stainings were evaluated manually and by using QuPath 0.2.3. The level for statistical significance was determined at *p* < 0.05. The size of the flaps and the malperfused/necrotic areas were analyzed using Image J.exe. All statistical analyses were performed using Microsoft Excel (Microsoft, Redmond, WA, USA) and Prism 9 (GraphPad Software, San Diego, CA, USA). 

The clinically visible necrotic area and the flap size were analyzed as follows: for the comparison of the medians of the same group at different timepoints, the Friedman test was used for not normally distributed samples and the one way-ANOVA test for normally distributed samples. Furthermore, comparison of different groups at the same timepoint was performed using the Brown Forsythe and Welch ANOVA test in normally distributed samples and the Kruskal Wallis test in not normally distributed samples. 

The following tests were used for the analyses of the stainings and of the PCR: first, QQ plots were used to identify the normal distribution graphically. For the comparison of the necrosis, the CD68+ cells and the counted vessels and ΔΔCt in different groups, an ordinary one-way ANOVA following the Tukey test was used when the samples were normally distributed and the Kruskal Wallis test was used when the samples were not normally distributed. Calculating 2^−ΔΔCt^ revealed the relative expression to previously operated non-irradiated control group [21]. 

## 3. Results

All animals tolerated the irradiation procedure and the operation. The results of the no cell group have been described previously [20]. 

**Figure 3:**
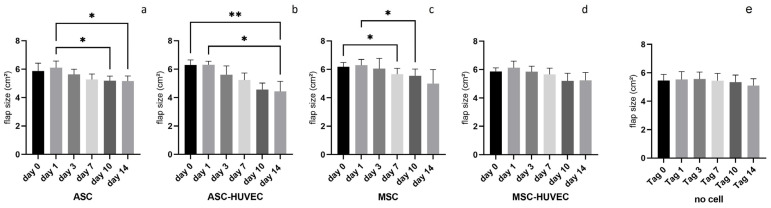
Reduction of the mean size of the irradiated right flaps in all groups from day 0 to day 14: (**a**) ASC; (**b**)ASC+HUVC; (**c**) MSC; (**d**) MSC+HUVEC; (**e**) no cell group; * = *p* < 0.05; ** = *p* < 0.01; *n* = 5 per group.

A reduction of the flap size of the right irradiated flaps (Figure 3) was measured in all groups from day 0 (operation) to day 14 (explantation). 

In the ASC group, mean flap size was reduced from 5.9 cm^2^ on day 0 to 5.2 cm^2^ on day 14, but without statistical significance. In the ASC-HUVEC group, comparison of the mean flap size from day 0 (6.3 cm^2^) to day 14 (4.4 cm^2^) showed a statistically significant reduction (*p* < 0.01). The reduction of the mean flap size in the MSC group from day 0 (6.2 cm^2^) to day 14 (5.0 cm^2^) was not statistically significant. In the MSC-HUVEC group, comparison of the mean flap size from day 0 (5.9 cm^2^) to day 14 (5.2 cm^2^) showed no statistically significant difference. The previously operated no cell group showed reduction of the flap size from 5.5 cm^2^ on day 0 to 5.1 cm^2^ on day 14. 

**Figure 4:**
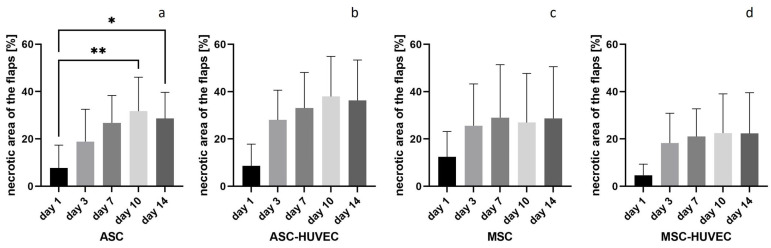
Increase in clinically visible necrotic area of the irradiated right flaps: (**a**) ASC; (**b**) ASC-HUVEC; (**c**) MSC; (**d**) MSC-HUVEC; * = *p* < 0.05; ** = *p* < 0.01; *n* = 5 per group.

The clinical visible mean necrotic area of the irradiated right flaps (Figure 4) increased in all groups from day 1 to day 14, as no necrotic area was seen on day 0 during the operation. 

In the ASC group, the necrotic area increase was statistically significant (*p* < 0.01) from 7.7% on day 1 to 28.6% on day 14. In the ASC-HUVEC group, the necrotic area increased from 8.6% on day 1 to 36.3% on day 14, but without statistical significance. In the MSC group, the increase in the necrotic area from 12.5% on day 1 to 28.7% on day 14 was not statistically significant. The necrotic area of the flaps in the MSC-HUVEC group increased from day 1 (4.7%) to day 14 (22.4%) without statistical significance. There was no statistically significant difference neither between the right flaps of the different groups on days 1, 7 and 14 nor between the right and left flaps of each group on these days. Comparison of the right flaps to a previously operated group that received 20 Gy preoperative irradiation without application of cells showed no difference. 

In the HE staining, mean percentage of the necrotic area of the right flaps was 18.0% in the ASC group, 32.3% in the ASC-HUVEC group, 27.1% in the MSC group and 25.5% in the MSC-HUVEC group. There was no statistically significant difference neither between the right flaps of the different groups nor between the right and left flaps of each on these days. Compared to a previously irradiated group that received 20 Gy preoperative irradiation alone (17.0%), no difference was found in all groups. 

Measurement of CD68-stained cells of the right flap showed a mean cell number per mm^2^ of 26.9 in the ASC group, 26.4 in the ASC-HUVEC group, 31.4 in the MSC group and 33.9 in the MSC-HUVEC group. 

**Figure 5:**
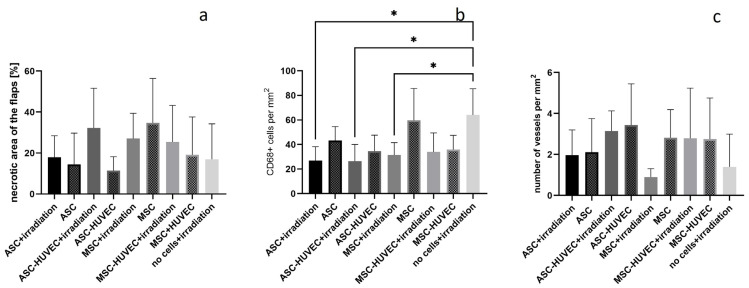
Difference in the irradiated right flaps: (**a**) percentage of necrotic area in the HE staining; (**b**) number of CD68-stained cells per mm^2^; (**c**) number of vessels per mm^2^; * = *p* < 0.05, *n* = 5 per group. Statistics showing the differences between the irradiated flaps of all groups and between the left and right flaps of each group.

There was no statistically significant difference neither between the right flaps of all groups nor between the right and left flaps of each group. Compared to a previously operated group (64.1 CD68 stained cells per mm^2^) that received 20 Gy preoperative irradiation without cell application [21], the ASC group, the MSC group as well as the ASC-HUVEC group showed a significantly lower number of CD68-stained cells (*p* < 0.05). The mean number of vessels per mm^2^ of the right flap was 2.0 in the ASC group, 3.1 in the ASC-HUVEC group, 0.9 in the MSC group and 2.8 in the MSC-HUVEC group (see all results in Figure 5). 

There was no statistically significant difference neither between the right flaps of all groups nor between the right and left flaps of each group nor compared to the no cells group (1.4). 

The relative expression of IL-6 in the irradiated right flaps in the ASC group was 0.42, 0.25 in the ASC-HUVEC group, 0.26 in the MSC group and 0.38 in the MSC-HUVEC group. There was no statistically significant difference neither between the right flaps of all groups nor between the right and left flaps of each group nor between the group that received 20 Gy preoperative irradiation in a previous study (0.94). 

Relative expression of HIF-1α in the irradiated right flaps in the ASC group was 15.9, in the ASC-HUVEC group 7.1, in the MSC group 6.2 and in the MSC-HUVEC group 7.7. The relative expression was statistically significantly lower in the MSC group compared to that in the ASC group. There were no further statistically significant differences neither between the right flaps of all groups nor between the right and left flaps of each group except in the ASC group (left flap 26.8, *p* < 0.05). Compared to the no cell group (3.5), the right flaps of the ASC group had a statistically significant higher relative expression of HIF-1α (*p* < 0.01). 

**Figure 6:**
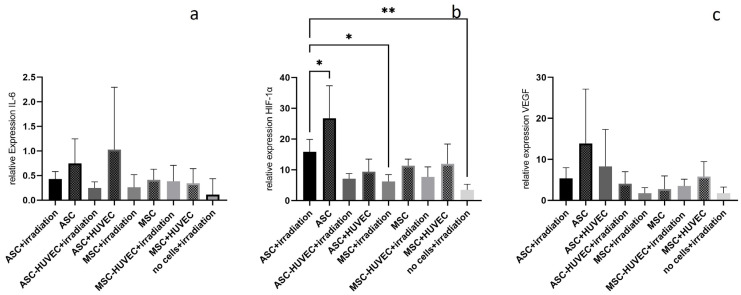
Relative expression in the irradiated right flaps compared to a control group without irradiation and without cells of (**a**) IL-6, (**b**) HIF-1α, (**c**) VEGF of the irradiated right flaps of each group; * = *p* < 0.05; ** = *p* < 0.01, *n* = 5 per group. Statistics showing the differences between the irradiated flaps of all groups and between the left and right flaps of each group.

Relative expression of VEGF in the irradiated right flaps in the ASC group was 5.4, 4.1 in the ASC-HUVEC group, 1.8 in the MSC group and 3.5 in the MSC-HUVEC group. There was no statistically significant difference neither between the right flaps of all groups nor between the right and left flaps of each group nor between the group that received 20 Gy preoperative irradiation alone (1.8) (see all results in Figure 6).

## 4. Discussion

The aim of this study was to investigate the influence of different cells on previously irradiated random pattern flaps. 

We noticed a reduction in the flap size in all groups from day 1 to day 14 with a statistically significant difference in the ASC and ASC-HUVEC groups. This shrinking of flaps in context with irradiation has already been described [20]. A reason might be that the irradiated skin has decreased elasticity and high-dose irradiation causes fibrosis [22]. We did not measure a reduction in the flap size in the no cell group. A possible explanation might be that the previously operated Lewis rats are not as sensitive to irradiation compared to the immunosuppressive RNU rats. Furthermore, the clinically visible necrotic area increased in all groups from day 1 to day 14. One reason for this is that the necrotic tissue is visible after cell death that takes hours to days depending on the tissue type [23]. 

The ASC, ASC-HUVEC and MSC groups showed a lower number of CD68-stained cells compared to the group that received 20 Gy preoperative irradiation, but without additional application of cells. These positive effects might be due to the fact that ASCs and MSCs have immunomodulatory, predominantly antiinflammatory activity [15]. Both ASCs and MSCs had immunosuppressive properties in vitro [24]. Furthermore, the number of vessels in the irradiated right flaps did not show significant differences between the cell groups and the group without cell application. Nevertheless, there was a trend towards a slightly higher number of vessels in the ASC, ASC-HUVEC and MSC-HVUEC groups compared to the MSC and no cell groups. ASCs promote neovascularization by enhancing angiogenesis and vasculogenesis [6]. This effect has been described in other studies. Implantation of fibrin microbeads containing ASCs and MSCs in a mouse hindlimb model improved limb salvage compared to that in the control group [18]. Furthermore, delivery of MSCs and HUVECs lead to the formation of microvascular networks throughout the implants [18]. Injection of a mixture of MSCs and HUVECs in a mouse model resulted in the formation of more robust and more mature vessels compared to injection of either cell type alone [19]. A reason might be a possible cooperation, interaction and crosstalk between two cell types in general [19]. 

The PCR analyses did not reveal differences in the expression of IL-6 and VEGF between the cell groups and the no cell group. But expression of HIF-1α was higher in the ASC group compared to both the MSC and no cells groups. An explanation might be that the expression of HIF-1α stimulates proliferation and angiogenic growth factors [25,26,27]. Human ASCs that were cultured under 2% O_2_ showed an increased proliferation rate compared to those cultured under ~21% O_2_. These findings were associated with the increased expression of HIF-1α [26]. The effect of ASCs on the expression of HIF-1α is even higher without the negative influence of the irradiation; the expression of HIF-1α in the non-irradiated left flaps treated with ASCs was even higher compared to that in the irradiated right ones. 

Nevertheless, the application of cells did not have an influence on the percentage of the necrotic area in irradiated flaps compared to that in the flaps that did not receive cell application [20,21]. In contrast to our results, the local application of ASCs between the flap and the wound resulted in a significantly higher average skin flap survival compared to that in the control group [28]. In our study, the cells were not injected directly into the flap; they had to migrate from the wound bed into the flap itself. Fibrin was chosen as the carrier substance due to its ability to promote wound healing and neovascularization and support angiogenesis [16,29]. A beneficial effect of this treatment has already been shown; stem cells delivered in fibrin spray have been shown to accelerate wound healing [30]. Furthermore, the implantation of fibrin microbeads containing ASCs and MSCs in a mouse hindlimb model improved limb salvage compared to that in the control group [18]. 

Other studies have shown a beneficial effect of MSCs or ASCs on flap survival after application in different ways. For example, subcutaneous administration of ASCs resulted in a higher mean percentage of flap survival area compared to that in the control group in the rat skin free flap model [31]. Moreover, ASCs were locally injected and increased the vital area of random pattern flaps [32,33,34]. Intravenous injection of mesenchymal stem cells promoted the skin survival of random pattern flaps that was associated with increased blood perfusion [35]. 

For successful stem cell therapy, the stem cells have to invade their target tissue [28,36]. A reason why the applied cells did not increase the viable area of the flaps might be an insufficient release of the cells from the fibrin glue. When testing different administration routes for cells in a similar flap model, the ASCs in the fibrin glue group were mainly attached to the flap bed and not found within the flap [37]. We administered the cells via the wound bed, as we expect an even distribution across the whole flap with continuous absorption. Furthermore, injection of stem cells into the tissue might result in uncontrolled distribution. 

In this study, we focused mainly on the comparison between the different treatment groups and not between the right and left flaps of each group. Intraindividual comparison is difficult in this setting as an influence of irradiation on non-irradiated areas as non-targeted bystander or distant effect cannot be excluded [38]. Cells that were exposed to ionizing radiation could release signals that induce very similar effects in non-irradiated neighboring cells [39]. The bystander effect in this context refers to the fact that responses in unirradiated cells were detected that might have occurred as a result of the exposure of other cells to IR [40]. To exclude a possible bystander effect definitely, no intraindividual control would have been possible as just one flap per rat could have been harvested. Furthermore, double the number of animals would have been needed.

This study has some limitations. In general, it takes a month until chronic irradiation-induced fibrosis has completely developed [2]. In this study, we focused on the optimization of early flap perfusion. Due to the shorter time between irradiation and surgery, a direct translation into daily clinical practice is limited. Nevertheless, it offers insights into a possible mechanism to improve flap perfusion at an early stage.

## 5. Conclusions

This study presents a feasible model to investigate the influence of cell application on irradiated random pattern flaps. Application of ASCs, MSCs and ASCs-HUVECs has an antiinflammatory effect. Treatment with MSCs and MSCs-HUVECs prevented early shrinking of the flaps and should be preferred in this context. Furthermore, a potential influence of cells on neovascularization has been noted.

## Figures and Tables

**Table 1:** Groups, IR = ionizing radiation, ASC = adipose mesenchymal stem cell, MSC = bone marrow-derived stem cell, HUVEC = human umbilical vascular endothelial cell.

Group	Right Flap	Left Flap
1	20 Gy IR + ASC	ASC
2	20 Gy IR + ASC + HUVEC	ASC + HUVEC
3	20 Gy IR + MSC	MSC
4	20 Gy IR + MSC + HUVEC	MSC + HUVEC

**Table 2:** Division of the flap for staining and PCR.

	Staining	PCR
Right/left flap	Cranial lateral third	Cranial medial third

**Table 3:** Primers including the housekeeping gene GAPDH.

Gene	5′-3′ Primer Sequence
GAPDH	For: GAAGGTCGGTGTGAACGGAT Rev: TGAACTTGCCGTGGGTAGAG
Interleukin 6	For: GACTTCCAGCCAGTTGCCTT Rev: GCAGTGGCTGTCAACAACAT
HIF-1α	For: GCAACTGCCACCACTGATGA Rev: GCTGTCCGACTGTGAGTACC
VEGF	For: AATGATGAAGCCCTGGAGTG Rev: ATGCTGCAGGAAGCTCATCT

## Data Availability

The data presented in this study are available on request from the corresponding author.
